# Supplementary material for: WO3−x/WS2 Nanocomposites for Fast-Response Room Temperature Gas Sensing
Source: Molecules. 2025 Jan 26;30(3):566. doi: 10.3390/molecules30030566 (PMC11820213; doi:10.3390/molecules30030566)
Supplement: Supplementary file 1 [file molecules-30-00566-s001.zip › molecules-3292940-supplementary.pdf]

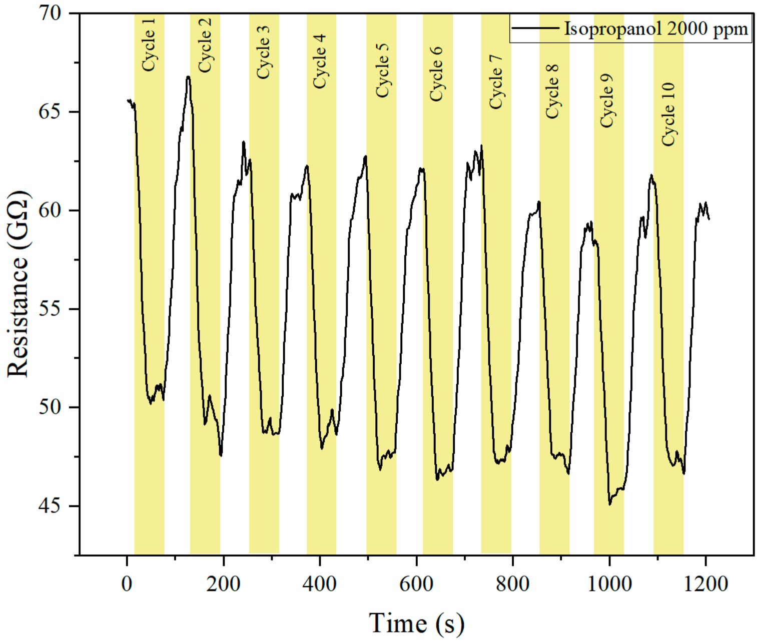

Figure S1. The number of duty cycles of the sensor when exposed to isopropanol (2000 ppm).

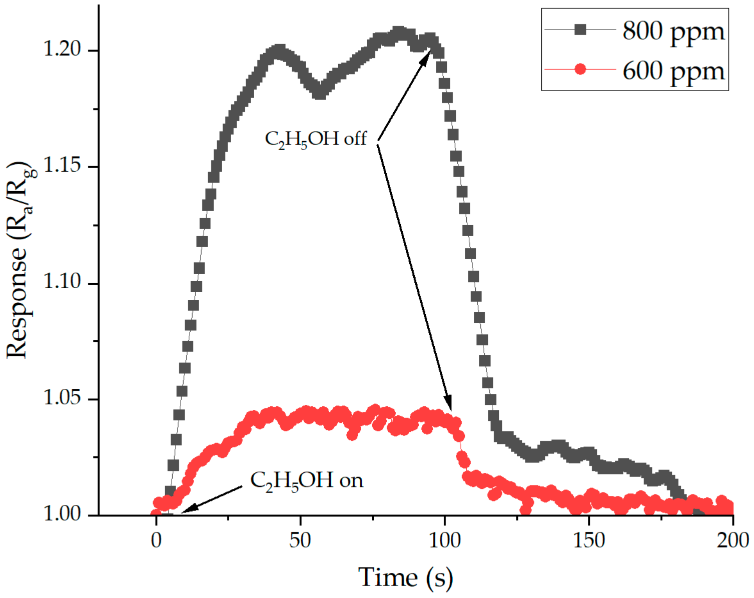

Figure S2. Time dependence of WO_{3-x}/WS_2 composite response to ethanol

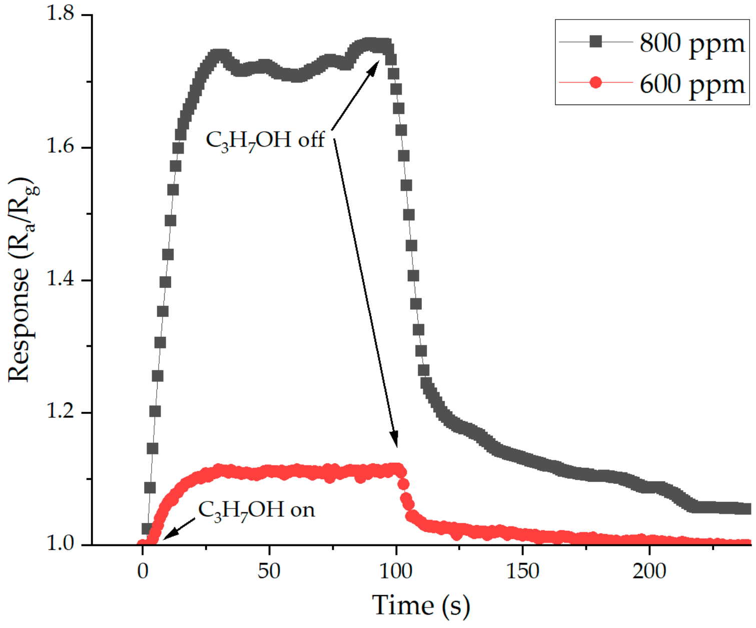

Figure S3. Time dependence of WO_{3-x}/WS_2 composite response to isopropanol

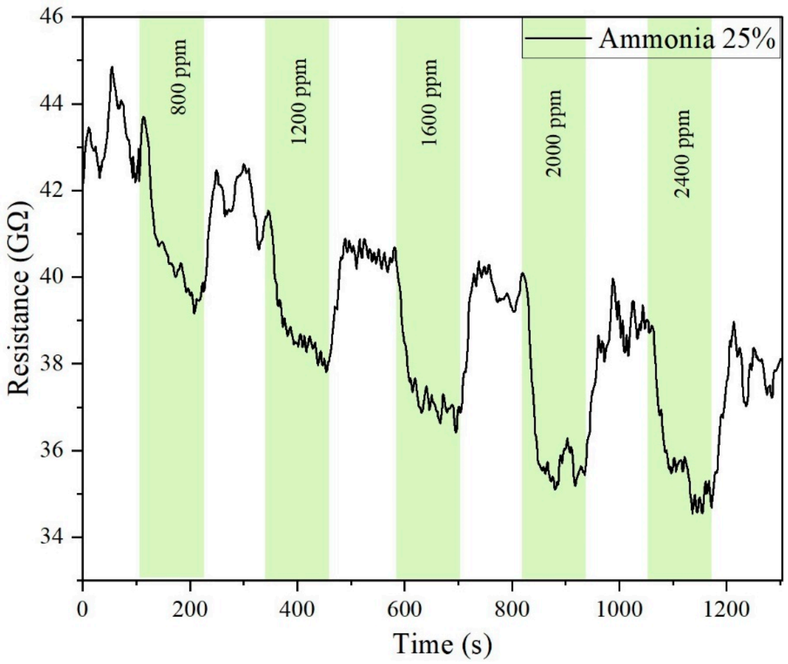

Figure S4. Sensor resistance when exposed toNH_3

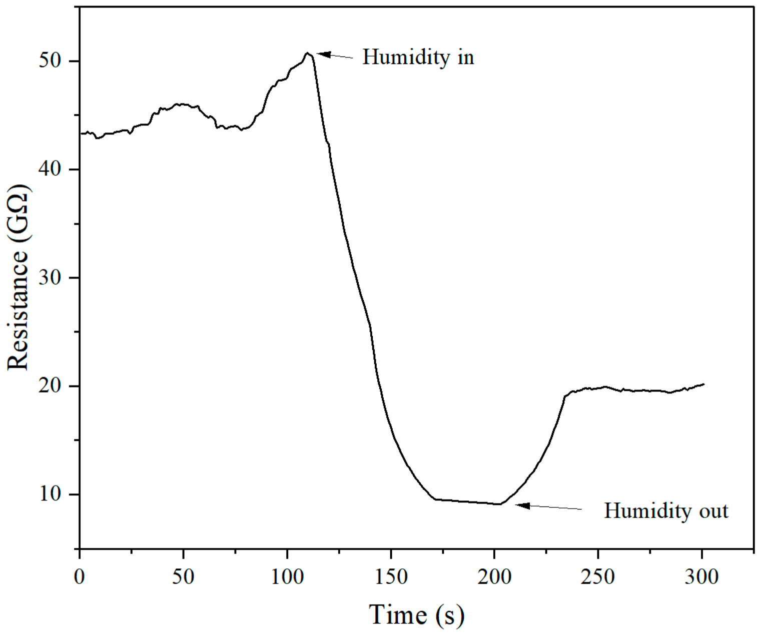

Figure S5. Change in sensor baseline resistance when exposed to humidity

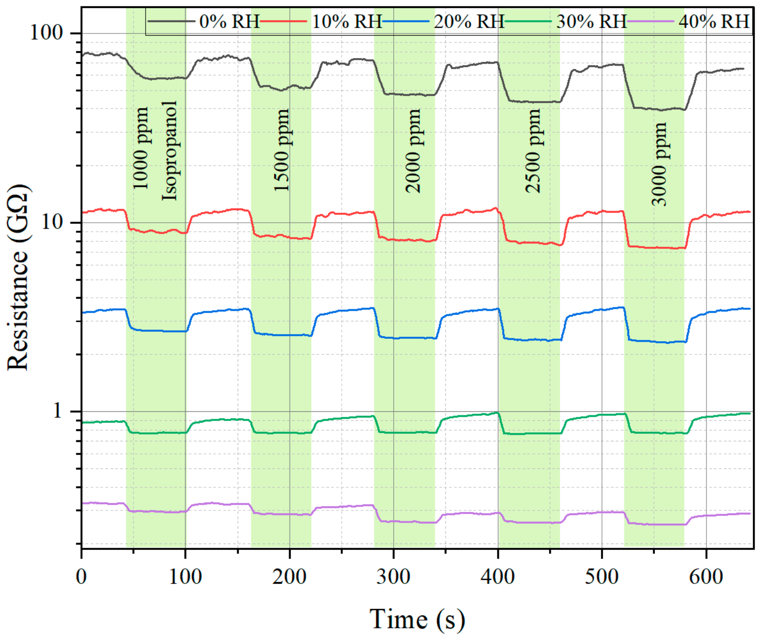

Figure S6. Influence of relative humidity on the sensor performance when changing isopropanol concentration
